# Harnessing novel engineered feeder cells expressing activating molecules for optimal expansion of NK cells with potent antitumor activity

**DOI:** 10.1038/s41423-021-00759-9

**Published:** 2021-09-27

**Authors:** Bokyung Min, Bitna Yang, You-Sun Kim, Gyeong Min Park, HyunAh Kim, Hyojin Kim, Eun-Ji Kim, Yu Kyeong Hwang, Eui-Cheol Shin, Sungyoo Cho

**Affiliations:** 1 BioMedical Science and Engineering Interdisciplinary Program, KAIST, Daejeon, Korea; 2 Cell Therapy Research Center, GC LabCell, Yongin, Korea; 3 Laboratory of Immunology and Infectious Diseases, Graduate School of Medical Science and Engineering, KAIST, Daejeon, Korea

**Keywords:** Immunotherapy, Cancer immunotherapy

Natural killer (NK) cells play an important role in the antitumor immune response as the major cytotoxic lymphocytes in the innate immune system. NK cells without any genetic modifications have been used for both autologous and allogeneic therapies. Moreover, many reports have suggested that allogeneic NK cells can reduce the recurrence of disease and improve survival through a graft-versus-leukemia effect without GVHD [1, 2].

For the successful clinical application of allogeneic NK cells, it is essential to be able to culture high-purity and high-efficacy NK cells in large quantities. Several groups have used autologous or allogenic peripheral blood mononuclear cells (PBMCs) as feeder cells for NK cell expansion [3, 4]. However, it is not clear which properties of PBMC feeder cells are important for NK cell proliferation. To address these questions, CD3^+^-depleted cells were cultured with γ-irradiated feeder cells, including whole PBMCs, purified CD14^+^ monocytes, and purified CD3^+^ T cells. We confirmed that direct contact between CD3^+^-depleted cells and PBMC feeder cells was important for the proliferation of NK cells (Supplementary Fig. S1A), and CD3^+^ T cells were found to be important for the expansion and cytotoxicity of NK cells, whereas CD14^+^ monocytes had little effect on NK cell expansion (Supplementary Fig. S1B). Among the feeder cell types, CD4^+^ T cells contributed to the highest NK cell proliferation, but NK cells showed similar cytotoxicity regardless of the feeder cell type in all conditions (Supplementary Fig. S1C). NK cells express many kinds of costimulatory and adhesion receptors, but with the exception of CD16, none of the receptors have been reported directly as key factors that influence human NK cell proliferation [5]. In our study, we performed blocking assays for costimulatory molecules such as 4-1BB, CD30, OX40, mTNF-α, and CD27, which are upregulated during NK cell expansion (Supplementary Fig. S2A, B). In blocking assays, we demonstrated that NK cell proliferation was inhibited by blocking 4-1BB and mTNF-α (Fig. 1A). In particular, 4-1BB blockade not only reduced NK cell proliferation but also decreased the expression of CD16, NKG2D, CD62L, and CXCR3, which are known to be important to NK cell function (Supplementary Fig. S3A, B).

**Fig. 1:**
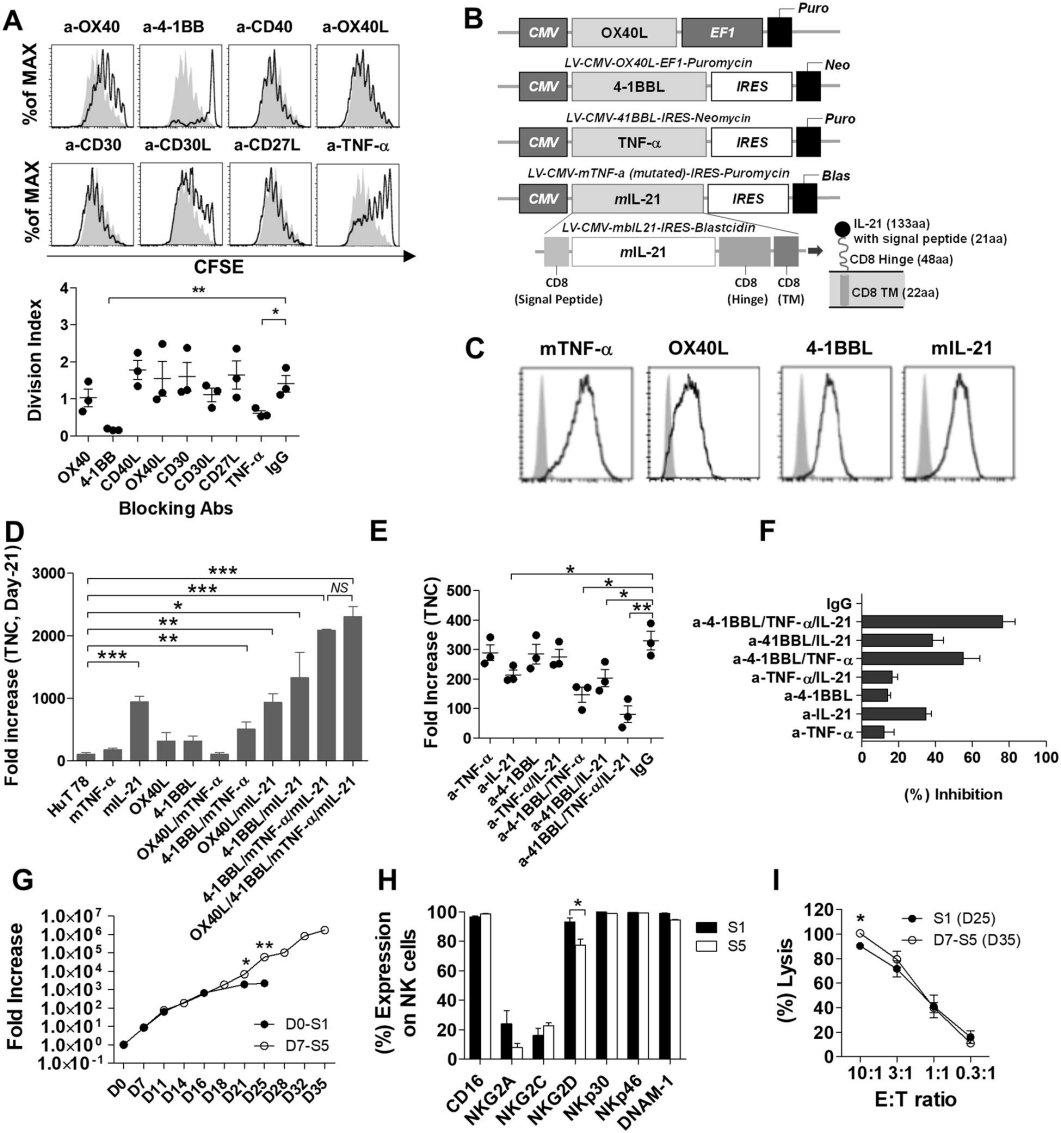
Development of eHuT 78 cells expressing 4-1BBL, mTNF-α, and mIL-21 for ex vivo NK cell expansion. **A** Costimulatory molecules important for NK cell proliferation. CFSE-labeled CD3^+^-depleted seed cells were treated with blocking antibodies as described and cultured for 7 days. The solid black line represents cells treated with blocking antibodies, while the filled gray peak represents cells treated with mouse IgG. Data are expressed as the mean ± SE (*n* = 3). *p < 0.05; **p < 0.01. **B** Scheme for the establishment of genetically engineered T cell lines. The HuT 78 cell line was transduced with lentiviral vectors containing different genes (OX40L, 4-1BBL, mTNF-α, and mIL-21). **C** The expression of each gene on transduced HuT 78 cells was measured by flow cytometry. **D** CD3^+^-depleted cells were cultured with HuT 78 cells expressing 11 different combinations of 4-1BBL, mTNF-α, and mIL-21 for 21 days. On day 21 of culture, fold increase was assessed. **E**, **F** To block eHuT 78 cells, cells were treated on day 0 with blocking antibodies specific as described. On day 14 of culture, the fold increase and the percentage of inhibition were assessed. Data are expressed as the mean ± SE (*n* = 3). *p < 0.05; **p < 0.01; ***p < 0.001. **G** CD3^+^-depleted cells were expanded by repeated stimulation with eHuT 78 cells every 7 days. The fold increase in cells stimulated once (S1) or five times (D7-S5) with eHuT 78 cells was assessed. **H** Phenotypes of expanded NK cells stimulated once (S1) or five times (D7-S5) with eHuT 78 cells were analyzed at 25 and 35 days of culture. **I** In vitro efficacy of expanded NK cells stimulated once (S1) or five times (D7-S5) with eHuT 78 cells was measured at 25 and 35 days of culture, respectively. The cytotoxicity of expanded cells against K562 cells was measured at E:T ratios of 10:1–0.3:1

The feasibility of using T cell lines instead of primary CD4^+^ T cells as feeder cells to expand NK cells was tested using seven different human T lymphoma cell lines. On day 14, the fold expansion and viability of NK cells in culture with HuT 78 was comparable to that of cells cultured with PBMC feeder cells (Supplementary Fig. S4A, B). We also checked the NK cell purity after 14 days of expansion. Contamination by CD3^+^, CD14^+^, and CD19^+^ cells was not significant in the culture with HuT 78, and the percentages of CD3^–^CD56^+^ and CD16^+^CD56^+^ NK cells in the culture with HuT 78 were near 100% (Supplementary Fig. S4C).

To induce continuous NK cell proliferation through repeated stimulation of HuT 78 cells, genes such as OX40L, 4-1BBL, mTNF-α, and mIL-21, which encode ligands that have previously been identified as capable of stimulating major NK cell proliferation factors, were transduced (Fig. 1B). The expression of transduced genes was confirmed by flow cytometry (Fig. 1C). We established several HuT 78 cell lines transduced with various combinations of the four genes and used them as feeder cells for NK cell expansion. In the present study, we selected 4-1BBL/mTNF-α/mIL-21-HuT 78 as the best new feeder cell candidate for NK cell expansion because OX40L/4-1BBL/mTNF-α/mIL-21-HuT 78 cells showed no more significant benefit than 4-1BBL mTNF-α/mIL-21-HuT 78 cells for NK cell proliferation (Fig. 1D). To confirm the effects of the transduced genes, we assessed NK cell proliferation after blocking each gene in a coculture of CD3^+^-depleted seed cells and 4-1BBL/mTNF-α/mIL-21-HuT 78 (eHuT 78). Blocking IL-21 alone or two costimulatory molecules at the same time significantly inhibited NK cell proliferation compared to blocking with mouse IgG. In particular, blocking all three genes inhibited NK cell proliferation by 80% or more (Fig. 1E, F).

To assess the feasibility of large-scale expansion of NK cells using eHuT 78, NK cells were cultured through repeated stimulation with eHuT 78 every 7 days. We compared the characteristics of NK cells stimulated with eHuT 78 once (S1) and NK cells stimulated with eHuT 78 five times (S5). S5 showed more than 700-fold expansion compared to S1 (Fig. 1G). An analysis of the immunophenotypes of S1 and S5 on the last day of culture showed similar expression of all markers except NKG2D, which appeared to be slightly higher in S1 than in S5 (Fig. 1H). Cytotoxicity against K562 at an E:T ratio of 10:1 was significantly higher in S5 than in S1, but there were no significant differences between S1 and S5 at lower E:T ratios (Fig. 1I). CD107a expression and cytokine production against K562 did not differ between S1 and S5 (Supplementary Fig. S5A). In this study, we showed that repeated stimulation with eHuT 78 robustly expanded NK cells while maintaining the expression of major proliferating and activating factors in NK cells and further showed that such explosively proliferated NK cells produce cytokines and exert cytotoxic function against tumor cells. In addition, we assessed the in vivo antitumor efficacy of NK cells cultured with eHuT 78 cells (PB-eHuT 78) in the Raji lymphoma model with or without rituximab. PB-eHuT 78 showed enhanced in vivo antitumor efficacy, especially with rituximab (Supplementary Fig. S5B). Therefore, we suggest that repeated stimulation by eHuT 78 as feeder cells can efficiently expand NK cells in large numbers and with excellent antitumor efficacy.

In conclusion, the use of eHuT 78 cells, which are T cell-based genetically engineered feeder cells, can be considered a core technology for anticancer NK immune cell therapy.

## Supplementary information

Fig. S1 Mode of action of PBMC feeder cells for NK cell expansion

Fig. S2. Identification of costimulatory molecules important for NK cell proliferation

Fig. S3. Inhibition of NK cell expansion by blocking 4-1BB and TNF-α signals for 14 days of culture

Fig S4. Assessment of NK cell expansion using T cell lines as feeder cells

Fig S5. In vitro and in vivo characteristics of NK cells expanded with eHuT 78 cells

Materials and Methods
