# Supplementary material for: Harnessing novel engineered feeder cells expressing activating molecules for optimal expansion of NK cells with potent antitumor activity
Source: Cell Mol Immunol. 2021 Sep 27;19(2):296–8. doi: 10.1038/s41423-021-00759-9 (PMC8803962; doi:10.1038/s41423-021-00759-9)
Supplement: Supplementary file 1 — Fig. S1 Mode of action of PBMC feeder cells for NK cell expansion [file 41423_2021_759_MOESM1_ESM.docx]

**Fig. S1**

**Fig. S1. Mode of action of PBMC feeder cells for NK cell expansion.** (A) Importance of cell-to-cell contact between CD3^+^-depleted seed cells and PBMC feeder cells during NK cell proliferation. CD3^+^-depleted cells were expanded with γ-irradiated PBMCs by cell-to-cell contact or no contact manner using transwells in the presence of 500 IU/mL IL-2 and 10 ng/mL OKT-3. After 14 days of culture, expanded NK cells were assessed for fold increase. (B) Comparison of PBMCs, CD3^+^ T cells and CD14^+^ monocytes as feeder cells for NK cell proliferation. CD3^+^-depleted cells were cultured with γ-irradiated PBMCs, purified CD3^+^ T cells or CD14^+^ monocytes in the presence of 500 IU/mL IL-2 and 10 ng/mL OKT-3 for 14 days. After 14 days of culture, expanded NK cells were assessed for fold increase (left) and *in vitro* cytotoxicity against K562 (right). The cytotoxicity of NK cells was measured at E:T ratios of 3:1 to 0.3:1. (C) Comparison of PBMCs, CD3^+^ T cells, CD4^+^ T cells, CD8^+^ T cells, and no feeder cells, as feeder cells for NK cell proliferation. CD3^+^-depleted cells were cultured with γ-irradiated PBMCs, CD3^+^ T cells, CD4^+^ T cells, CD8^+^ T cells, or no feeder cells in the presence of 500 IU/mL IL-2 and 10 ng/mL OKT-3 for 14 days. After 14 days of culture, expanded NK cells were assessed for fold increase (left) and *in vitro* cytotoxicity against K562 (right). Data are expressed as mean + SE (n = 3). **p*<0.05; ***p*<0.01; ****p*<0.001.
