# Supplementary material for: Harnessing novel engineered feeder cells expressing activating molecules for optimal expansion of NK cells with potent antitumor activity
Source: Cell Mol Immunol. 2021 Sep 27;19(2):296–8. doi: 10.1038/s41423-021-00759-9 (PMC8803962; doi:10.1038/s41423-021-00759-9)
Supplement: Supplementary file 2 — Fig. S2. Identification of costimulatory molecules important for NK cell proliferation [file 41423_2021_759_MOESM2_ESM.docx]

**Fig. S2**

**Fig. S2. Identification of costimulatory molecules important for NK cell proliferation.** (A-B) Expression of costimulatory molecules on NK cells during the entire culture period. CD3^+^-depleted cells were co-cultured with γ-irradiated PBMCs in the presence of 500 IU/mL IL-2 and 10 ng/mL OKT-3 for 23 days. The expression of costimulatory receptors and their ligands on NK cells was measured by flow cytometry from 0 to 23 days. Data were analyzed quantitatively by FlowJo software.
