# Supplementary material for: Harnessing novel engineered feeder cells expressing activating molecules for optimal expansion of NK cells with potent antitumor activity
Source: Cell Mol Immunol. 2021 Sep 27;19(2):296–8. doi: 10.1038/s41423-021-00759-9 (PMC8803962; doi:10.1038/s41423-021-00759-9)
Supplement: Supplementary file 3 — Fig. S3. Inhibition of NK cell expansion by blocking 4-1BB and TNF-α signals for 14 days of culture [file 41423_2021_759_MOESM3_ESM.docx]

**Fig. S3**

**Fig. S3. Inhibition of NK cell expansion by blocking 4-1BB and TNF-α signals for 14 days of culture.** CD3^+^-depleted cells were treated with anti-4-1BB mAb, anti-TNF-α mAb, or mouse IgG, and cultured with γ-irradiated PBMCs in the presence of 500 IU/mL IL-2 and 10 ug/mL OKT-3 for 14 days. (A) On day 14, the fold increase of expanded NK cells was determined by cell number before (day 0) and after (day 14) culture. (B) Immunophenotypes of expanded NK cells were measured by flow cytometry and analyzed by FlowJo. Data are expressed as mean ± SE (n = 3). * *p*<0.05; ** *p*<0.01.
