# Supplementary material for: Harnessing novel engineered feeder cells expressing activating molecules for optimal expansion of NK cells with potent antitumor activity
Source: Cell Mol Immunol. 2021 Sep 27;19(2):296–8. doi: 10.1038/s41423-021-00759-9 (PMC8803962; doi:10.1038/s41423-021-00759-9)
Supplement: Supplementary file 4 — Fig S4. Assessment of NK cell expansion using T cell lines as feeder cells [file 41423_2021_759_MOESM4_ESM.docx]

**Fig. S4**

**Figure S4. Assessment of NK cell expansion using T cell lines as feeder cells.** CD3^+^-depleted cells were cultured with various T cell lines as feeder cells in the presence of 500 IU/mL IL-2 and 10 ng/mL OKT-3 for 14 days. All T cell lines were γ-irradiated with 20,000 cGy immediately before co-culturing. (A) The fold increase and (B) cell viability were assessed using the propidium iodide staining method after 14 days of culture. (C) On day 14 of culture, the purity of NK cells (CD3^-^CD56^+^, CD16^+^CD56^+^, CD3^+^, CD14^+^, and CD19^+^ cells) was measured by flow cytometry and analyzed by FlowJo. Data are expressed as mean ± SE (n = 3). * *p*<0.05.
