# Supplementary material for: Harnessing novel engineered feeder cells expressing activating molecules for optimal expansion of NK cells with potent antitumor activity
Source: Cell Mol Immunol. 2021 Sep 27;19(2):296–8. doi: 10.1038/s41423-021-00759-9 (PMC8803962; doi:10.1038/s41423-021-00759-9)
Supplement: Supplementary file 5 — Fig S5. In vitro and in vivo characteristics of NK cells expanded with eHuT 78 cells [file 41423_2021_759_MOESM5_ESM.docx]

**Fig. S5**

**Figure S5. *In vitro and in vivo* characteristics of NK cells expanded with eHuT 78 cells. (A)** CD3^+^-depleted cells were expanded by repeated stimulation using eHuT 78 cells every 7 days. Seed cells were stimulated once (S1) or five times (D7-S5) with eHuT 78 cells. CD107a, IFN-γ and TNF-α expression of expanded NK cells against K562 was measured at an E:T ratio of 1:1. Data are expressed as mean ± SE (n = 3). (B) To assess the *in vivo* anti-tumor activity of NK cells expanded with eHuT 78 cells (PB-eHuT 78), CD3^+^-depleted cells were expanded by stimulation twice using each feeder cells for 3 to 4 weeks. SCID mice injected intravenously with 1 × 10^5^ Raji cells on day 0 were administered freezing media (0.2 mL) plus human IgG (0.01 µg), rituximab alone (0.01 µg), or expanded/cryopreserved NK cells stimulated with PB-eHuT 78 (2 × 10^7^ cells) with or without rituximab. Freezing media and expanded/cryopreserved NK cells were administered intravenously on day 1, 2, 3, 6, 7, and 8, and rituximab and human IgG were injected subcutaneously on day 1. Survival was monitored. The experiment was performed using 10 mice per group.
