# Supplementary material for: Harnessing novel engineered feeder cells expressing activating molecules for optimal expansion of NK cells with potent antitumor activity
Source: Cell Mol Immunol. 2021 Sep 27;19(2):296–8. doi: 10.1038/s41423-021-00759-9 (PMC8803962; doi:10.1038/s41423-021-00759-9)
Supplement: Supplementary file 6 — Materials and Methods [file 41423_2021_759_MOESM6_ESM.docx]

**Supplemental Materials - Materials and Methods**

**Ethics statement**

Lymphapheresis samples were obtained following acquisition of the study participants’ written informed consent, in accordance with the Declaration of Helsinki. Cord Blood Units (CBU) were obtained from the Seoul Metropolitan Government Public Cord Blood Bank. The research protocols were reviewed and approved by the institutional review board of Seoul National University Hospital (Permit Number: H-1004-027-315) and the institutional review board of Green Cross LabCell (Permit Number: IRB008), respectively. All experiments were performed in accordance with the national guidelines for animal care in Korea. All animal studies were conducted according to the institutional guidelines established by GC Pharma Institutional Animal Care and Use Committee (Approval Number: GC-16-117A).

**Human cell lines**

H9, HuT 78, MOLT 3, MOLT 13, PEER, RPMI8402, TALL-01, K562, and Raji cells were purchased from American Type Culture Collection (ATCC). Cell lines were cultured in RPMI-1640 medium (GIBCO, USA) supplemented with 10% FBS (GIBCO, USA) and 2 mM L-glutamine (GIBCO, USA).

**Cell isolation**

CD3^+^ cells were removed using the VarioMACS (Miltenyi Biotec, Germany) magnetic sorting system for NK cell enrichment from healthy donor-derived PBMCs, which were collected via lymphapheresis. In some experiments, CD3^+^, CD4^+^, CD8^+^, and CD14^+^ cells were positively isolated using the MiniMACS (Miltenyi Biotec, Germany) magnetic sorting system to be used as feeder cells.

**Generation of genetically engineered T cell lines**

HuT 78 cells were transduced with OX40L, 4-1BBL, mTNF-α, or mIL-21, alone or in combination, respectively. The 4-1BBL insert was prepared from 4-1BB-expressing vector (Origene, USA) by PCR. OX40L was synthesized from Bioneer (South Korea). mIL-21 was synthesized with the sequence of IL-21 active protein, CD8 signal peptide, CD8 hinge, and CD8 transmembrane, and further codon optimized. cDNA of mTNF-α was prepared by reverse transcription-PCR (RT-PCR) from PBMCs. TNF-α−converting enzyme (TACE) recognition site mutation was introduced by replacing Ala-Val (A-V) with Pro-Val (P-V) using a site-directed mutagenesis kit (Agilent Technologies, USA). Inserted genes and lentiviral vectors (SBI, USA) were digested by EcoRI and BamHI (New England BioLabs, USA) and ligated using the In-Fusion HD cloning kit (Clontech, USA). Lentiviral concentrate was produced in 293T by lipofectamine 2000 (ThermoFisher Scientific, USA) and concentrated by Amicon Ultra-15 Centrifugal Filter Unit with Ultracel-100 membrane (Merckmillipore, USA). HuT 78 cells (0.5 × 10^6^ cells/mL) were suspended in 1 mL OPTI-MEM (ThermoFisher Scientific, USA) containing 50 μL lentiviral concentrate and 10 μg/mL polybrene (Santa Cruz Biotechnology, USA) and spinoculated at 1800g, 32°C for 90 minutes. HuT 78 cells transduced with the lentiviral system were selected with antibiotics. 4-1BBL/mTNF-α/mIL-21 positive HuT 78 cells were isolated by flow cytometry-guided sorting (FACSMelody^TM^ Cell Sorter, BD Bioscience, USA).

***Ex vivo* expansion and cryopreservation of NK cells**

*Ex vivo* expansion and cryopreservation of NK cells were performed as described previously (1-2). CD3^+^-depleted cells (1 × 10^6^ cells/mL) were seeded in CellGro SCGM medium (CellGenix, Germany) containing 1% ~ 2% donor plasma, γ-irradiated (2,000 rad) donor PBMCs (5 × 10^6^ cells/mL), 500 IU/mL IL-2 (Novartis, Switzerland) and 10 ng/mL anti-CD3 monoclonal antibody OKT3 (eBioscience, USA). Cultured cells were fed with CellGro SCGM containing 1% donor plasma and 500 IU/mL IL-2 (culture medium) to maintain a cell concentration of 1~2 × 10^6^ cells/mL for 14 days. For more than 21-day culture, restimulation of PBMC feeder cells was performed every 7 days. At this time, the cultured cells were suspended with the culture medium at a concentration of 1 × 10^6^ cells/mL and five-fold of γ-irradiated (2,000 rad) donor PBMCs (5 × 10^6^ cells/mL) and 10 ng/mL OKT3. In some experiments, γ-irradiated (2,000 rad) CD3^+^, CD4^+^, CD8^+^, or CD14^+^ cells were used as feeder cells instead of PBMCs. For the screening of cell lines for feeders, various types of T cell lines such as RPMI8402, MOLT 3, MOLT 13, TALL-01, PEER, HuT 78, and H9 cells, were γ-irradiated with 20,000 cGy and used as feeder cells. For NK cell culture with blocking antibody (Ab), 10 μg/mL anti-OX40 Ab (BD, USA), 10 μg/mL anti-4-1BB Ab (LSBio, USA), 30 μg/mL anti-TNF-α Ab (Janssen, Belgium), or 10 μg/mL mouse IgG (BD, USA), were added to each well on day 0, respectively. To prevent cell-to-cell contact, NK cells were cultured using a transwell (Corning, USA).

For expansion of NK cells using genetically modified HuT 78 cell lines, 4-1BBL/mTNF-α/mIL-21 transduced HuT 78 (eHuT 78) cells were γ-irradiated with 20,000 cGy. The ratio of CD3^+^-depleted seed cells (1 × 10^6^ cells/mL) to eHuT 78 feeder cells (2.5 × 10^6^ cells/mL) was 1 to 2.5. Depending on the culture duration, NK cells were stimulated with e-HuT 78 every 7 days for 1 to 5 times. To block signaling molecules during NK cell expansion with eHuT 78, 30 μg/mL anti-4-1BBL Ab (MYBiosource, USA), 10 μg/mL anti-TNF-α Ab (Janssen, Belgium), 10 μg/mL anti-IL-21 Ab (MABTECH, Sweden), and mouse IgG (R&D systems, USA), were added to the co-cultured cells at day 0 of culture. Cell count and viability were assessed by staining with propidium iodide using an automatic cell counter (Digital Bio, South Korea). Fold increase of expanded cells was calculated by dividing the number of live cells measured on a specific day of culture by the number of live seed cells on day 0 of culture.

For the comparison of expansion of cord blood-derived NK cells, CD3^+^-depleted CB cells (1 × 10^6^ cells/mL) were cultured with γ-irradiated (2,000 rad) donor PBMCs (5 × 10^6^ cells/mL), γ-irradiated (10,000 rad) HuT 78 (2.5 × 10^6^ cells/mL), and eHuT 78 (2.5 × 10^6^ cells/mL), in CellGro SCGM medium containing 2% donor plasma, 1000 IU/mL IL-2 and 10 ng/mL anti-CD3 monoclonal antibody OKT3. Depending on culture duration, eHuT 78 cells were stimulated every 2 weeks.

Cultured cells were fed with CellGro SCGM containing 1% donor plasma and 1000 IU/mL IL-2 (culture medium) to maintain a cell concentration of 1~2 × 10^6^ cells/mL for 14 or 28 days. For cryopreservation of NK cells, harvested cells were suspended in freezing media and stored in an LN2 tank (2).

**CFSE proliferation assays**

CD3^+^-depleted cells were labelled using CellTrace CFSE cell proliferation kit (ThermoFisher Scientific, USA) according to the manufacturer’s instructions. CD3^+^-depleted cells labelled with CFSE were seeded in 0.5 mL of CellGro SCGM medium (CellGenix, Germany) with 2% auto-plasma, irradiated (2,000 rad) donor PBMCs (2.5 × 10^6^ cells), 500 IU/mL IL-2 (Novartis, Switzerland), and 10 ng/mL anti-CD3 monoclonal antibody OKT3 (eBioscience, USA), in 12-well plates (Corning, USA). For the Ab blocking assay, 10 μg/mL of anti-CD27L Ab (Ancell, USA), anti-CD30 Ab (Ancell, USA), anti-CD30L Ab (R&D systema, USA), anti-OX40 Ab (BD, USA), anti-4-1BB Ab (LSBio, USA), anti-CD40L Ab (Biolegend, USA), anti-OX40L Ab (Ancell, USA), anti-TNF-α Ab (Janssen, Belgium), or MsIgG (BD, USA) were added to each well at 0 days of incubation. Cells were harvested at 7 days after culture. Fluorescence associated with CFSE gated on live CD3^-^CD56^+^ cells was measured by LSR Fortessa (BD, USA) and data were analyzed using FlowJo software (BD, USA).

**Immunostaining and flow cytometric analysis**

The following monoclonal antibodies were used to stain NK cells: anti-CD56-APC-eFluor^®^780 (CMSSB) (eBioscience, USA), anti-CD3-FITC (UCHT1), anti-CD14-FITC (M5E2), anti-CD16-PE (3G8), anti-DNAM-1-PE (DX11), anti-CD56-PE-Cy5 (B159), anti-NKp30-PE (P30-15), anti-NKp44-PE (P44-8.1), anti-NKp46-PE (9E2/NKp46), anti-OX40L-PE (ik-1), anti-4-1BBL-PE (C65-485), anti-4-1BB-PE (C65-485), anti-OX40-PE (ACT35), anti-CD27-PE (MT-271), anti-CD27L-PE (Ki-24), anti-CD30-PE (BerH8), anti-CD30L-PE, anti-CD3-PE-cy5.5 (SP34-2), anti-CD4-FITC (RPA-T4) (BD Biosciences, USA), anti-NKG2A-PE (131411), anti-NKG2C-PE (134591), anti-NKG2D-PE (149810), anti-TNF-α (membrane)-PE (6401), anti-TNFRII-PE (22235) (R&D systems, USA), and anti-CD30L-PE (RM153) (Biolegend, USA). Live cells were gated with 7-AAD (Beckman-Coulter, USA). eHuT 78 cells were stained with anti-TNF-α (membrane)-PE (6401) (R&D systems, USA), anti-OX40L-PE (ik-1), anti-4-1BBL-PE (C65-485) (BD Biosciences, USA), and anti-IL21-PE (3A3-N2) (eBioscience, USA). Stained cells were acquired on LSR Fortessa and data were analyzed using FlowJo software (TreeStar Inc., OR).

***In vitro* cytotoxicity assays**

Cytotoxicity of NK cells against tumor target cell lines was assessed by fluorometric cytotoxicity assay. Tumor cells were stained with 30 mM calcein-AM (Molecular probe, USA) for 1 hour at 37℃. NK cells were prepared at effector: target (E:T) ratios of 10:1 to 0.3:1. NK cells and labeled tumor target cells were co-cultured in 96-well plates in triplicates at corresponding ratios at 37℃ and 5% CO_2_ for 4 hours with light-protection. RPMI1640 medium containing 10% FBS or 0.1% triton-X100 was added to the targets to provide spontaneous and maximum release. Measurement was conducted at excitation 485 nm and emission 535 nm with the fluorometer. The percentage of specific calcein AM release was calculated according to the formula: % specific release = [(mean experimental release−mean spontaneous release)/(mean maximal release−mean spontaneous release)]×100.

**Intracellular cytokine and CD107a staining**

To measure intracellular cytokines and CD107a of NK cells, NK cells were co-cultured with tumor targets at a 1:1 ratio for 4 hours in the presence of anti-CD107a-APC (H4A3; BD Biosciences, USA), GolgiStop™ and GolgiPlug™ (BD Biosciences, USA). After 4 hours, cells were washed with BD FACS flow buffer and stained with anti-CD3-FITC, anti-CD56-APC-eFluor^®^780, and 7-AAD permeabilized by BD CytoFix/CytoPerm™, and then stained with anti-IFN-γ-PE (B27; BD Biosciences) and anti-TNF-α-PE-Cy7 (Mab11; eBioscience). Stained cells were acquired on LSR Fortessa and data analysis was conducted using FlowJo software (TreeStar Inc., OR).

***In vivo* mouse study**

The Raji lymphoma mouse model was used to test the anti-tumor efficacy of NK cells. Six-week-old female CB-17-Prkdc^Scid^ mice (Charles River, Japan) were inoculated in the tail vein with Raji cells (1 × 10^5^ cells) at day 0. Rituximab (Roche, Switzerland) or human IgG (Sigma, USA) was administered subcutaneously into the mice at day 1. Expanded/cryopreserved NK cells (2 × 10^7^ cells) or freeze media were administered intravenously at days 1, 2, 3, 6, 7, and 8. Expanded/cryopreserved NK cells were thawed at 37℃ in a water bath and injected immediately into the mice. Individual mice were monitored daily for tumor-associated paralysis and survival. All animal experiments were performed according to the national guideline governing animal care in Korea.

**Statistical analysis**

Statistical significance for *in vitro* studies was determined using the unpaired student’s t-test. Mouse survival was analyzed by Kaplan-Meier method and statistical significance was calculated by log-rank test using GraphPad Prism software (GraphPad Software Inc., CA). A *P* value below 0.05 was used to show statistically significant differences for all tests.
**References**

1. Lim O, Lee Y, Chung H, Her JH, Kang SM, Jung M-y, et al. GMP-compliant, large-scale expanded allogeneic natural killer cells have potent cytolytic activity against cancer cells in vitro and in vivo. *PLoS one*. 2013;**8**:e53611.

2. Min B, Choi H, Her JH, Jung MY, Kim H-J, Jung M-y, et al. Optimization of large-scale expansion and cryopreservation of human natural killer cells for anti-tumor therapy. *Immune network*. 2018;**18**:e31.
